# False-belief reasoning from 3 to 92 years of age

**DOI:** 10.1371/journal.pone.0185345

**Published:** 2017-09-28

**Authors:** Daniel M. Bernstein, Alisha Coolin, Ashley L. Fischer, Wendy Loken Thornton, Jessica A. Sommerville

**Affiliations:** 1 Kwantlen Polytechnic University, Surrey, British Columbia, Canada; 2 Simon Fraser University, Burnaby, British Columbia, Canada; 3 University of Washington, Seattle, Washington, United States of America; University College London, UNITED KINGDOM

## Abstract

False-belief reasoning, defined as the ability to reason about another person’s beliefs and appreciate that beliefs can differ from reality, is an important aspect of perspective taking. We tested 266 individuals, at various ages ranging from 3 to 92 years, on a continuous measure of false-belief reasoning (the Sandbox task). All age groups had difficulty suppressing their own knowledge when estimating what a naïve person knew. After controlling for task-specific memory, our results showed similar false-belief reasoning abilities across the preschool years and from older childhood to younger adulthood, followed by a small reduction in this ability from younger to older adulthood. These results highlight the relative similarity in false-belief reasoning abilities at different developmental periods across the lifespan.

## Introduction

Successful perspective taking requires the ability to represent and reason about another’s beliefs and feelings. Central to social cognition and behavior across development, perspective taking relates to variability in outcomes such as academic achievement and socio-emotional adjustment [1–3]. Thus, understanding how perspective taking differs across ages is important. The current study focuses on one type of perspective taking: false-belief reasoning, defined as the ability to reason about another person’s beliefs and appreciate that beliefs can differ from reality. False-belief reasoning is a central component of theory of mind (ToM) [4]. While past research with children has revealed a significant shift in the ability to appreciate false beliefs during the preschool years, less is known about differences in false-belief reasoning from preschool to old age [5, 6].

There are two main obstacles to studying false-belief reasoning across the lifespan. First, most false-belief reasoning tasks require categorical responses, which may produce floor and ceiling effects. This obstacle can make continuous and quantitative differences appear categorical and qualitative. For example, in the classic ToM “change-of-location” task that measures false-belief reasoning, children hear a story involving Sally and Ann playing with a ball. Sally places the ball in a box and leaves the room. While Sally is away, Ann moves the ball to a cupboard. When Sally returns to the room, children must indicate where Sally will look for the ball (false-belief question) and where Sally put the ball before she left (memory-control question). Because there are only two response options to each of these questions, researchers then interpret performance on this task as all-or-none: The child either understands the concept of false-belief reasoning or doesn’t [7]. This task cannot be used with adolescents and adults because their performance tends to be at the task ceiling; however, much evidence regarding adult false-belief performance derives from tasks that categorize information-rich responses in a similarly reductionist fashion [8]. One way in which developmental psychologists have addressed the problem of using categorical tasks to measure false-belief reasoning is by combining performance across a battery of age-appropriate categorical tasks to yield a continuous measure of performance [9].

The second obstacle to studying false-belief reasoning across the lifespan is that, although individual differences in memory, language and executive function correlate with false-belief reasoning, in some studies age differences in these cognitive processes have failed to capture important individual differences in false-belief reasoning [10]. For example, researchers have shown significant associations between false-belief performance and separate tasks measuring episodic memory [11]. The correlation between memory and false-belief reasoning (based on separate measures) tells us that memory (as a general cognitive ability) is an important component of false-belief reasoning [12]; however, this correlation may not capture task-specific memory processes that one needs to perform false-belief reasoning tasks (see [13, 14]). We maintain that these task-specific processes may contribute additional age variance to false-belief performance that we would miss by correlating performance on a separate memory task with a false-belief reasoning task. Indeed, memory for task-specific information relies on many factors, such as participants’ motivation to recall the task information and/or to recall social information more broadly.

To address these obstacles, we need (1) continuous tasks to assess false-belief reasoning, and (2) to measure participants’ memory for information presented in the task itself to control for variance due to task-specific memory. Researchers have created a continuous false-belief task modeled on the classic change-of-location task, the Sandbox task [15]. Instead of using two discrete response options, the experimenters hid an object inside a large container while both characters were present, and then moved the object within the same container while the protagonist was away. When asked to predict where the protagonist would search for the hidden object upon her return (false belief) or where the protagonist initially placed the object (memory control), participants could indicate any location in the container. This task has demonstrated utility in documenting age differences in false-belief reasoning. Preschoolers and college students had difficulty suppressing their own knowledge to reason about another person’s false belief, but performance improved with age [15]. In follow-up work, middle-aged and older adults showed more difficulty with false-belief reasoning than did younger adults [16]. These results suggest that false-belief reasoning undergoes periods of gains and losses throughout the lifespan. The dynamic nature of false-belief reasoning is further supported by adult research showing that older adults routinely make more errors than younger adults on other tasks measuring false-belief reasoning [10].

We present the first study to examine differences in false-belief reasoning across a wide age span: throughout the preschool years, from older childhood to younger adulthood, and from younger to older adulthood. To accomplish this goal, we used a single continuous measure that controlled for participants’ ability to remember task-specific information.

## Materials and methods

### Participants

This study received Research Ethics Board approval from Kwantlen Polytechnic University and Simon Fraser University. Adult participants and parents of child participants provided written informed consent. Children provided oral assent. Participants were 266 individuals ranging from 3 to 92 years, and comprising five age groups: twenty-three 3-year olds (*M* = 41.00 months, *SD* = 4.16; 15 female), twenty 5-year olds (*M* = 62.30 months, *SD* = 2.81; 16 female), forty-one 9- to 12-year olds (*M* = 10.51, *SD* = 1.16, 23 female), one hundred and twelve 17- to 25-year olds (*M* = 19.2 years, *SD* = 1.76, 81 female, 4 missing gender information), and seventy 65- to 92-year olds (*M* = 72.8 years, *SD* = 5.66, 42 female).

This study was based in part on existing data; therefore, sample sizes varied across age groups: The 3- and 5-year olds’ data are from ([15]: Experiment 2). The remaining data are unpublished; however, some of the data and Methods are described elsewhere [17]. The 3- and 5-year olds came from a university-maintained child database. The 9- to 12-year olds came from elementary and middle schools. The younger adults were university students. The older adults were a healthy, community-dwelling sample screened for cognitive impairment (see [17] for description of older and younger adult samples and recruitment). All participants were fluent English speakers. (data are available for download in S1 Data; see S1 Text).

We note that not all ages within the 3–92 year age range are represented. The rationale for assessing false-belief reasoning at specific points along the lifespan, aside from convenience, was based on prior work. Specifically, we included the two preschool age groups based on work showing classic gains in false-belief reasoning between 3- and 5-year olds. We included the 9- to 12-year olds based on work showing surprisingly late specificity in right temporal-parietal junction associated with false-belief reasoning that roughly corresponds to these adolescent ages [18]. Finally, we included our adult samples based on work showing systematic decline in false-belief reasoning and other theory of mind measures from younger to older adulthood [10].

### Materials

The Sandbox task followed that of Sommerville et al. ([15]: Experiment 2), and involved a rectangular Styrofoam-filled box 60 inches long by 18 inches wide by 12 inches deep. The experimenter enacted nine stories, one per trial, each involving two characters and a hidden object. The physical Sandbox represented different things in each story (e.g., sandbox, bathtub, planter box). In eight of the stories (four false-belief trials, four memory-control trials), a protagonist placed a small object in the Sandbox at one location (L1) and exited the scene. While the protagonist was away (false-belief and memory-control trials), a second character moved the object to another location (L2) within the Sandbox. Participants received one critical question at the end of each trial: False-belief trials required participants to take the perspective of the protagonist, who had a false belief about the object’s location; memory-control trials required participants to remember the object’s initial location. In one story (true-belief trial), the protagonist watched as the other character moved the object. The purpose of this trial was to prevent participants from realizing that the correct response on every trial was L1 (true belief correct response was L2). Relative to the two other conditions, all age groups showed significantly more bias toward L2 on the true-belief trial. We do not consider the true-belief trial further.

As in [15], participants completed a 20-second visual search filler task prior to receiving the critical question. For false-belief and memory-control trials, we varied whether L2 was to the right or left of L1. The distance between L1 and L2 was 14 inches on all trials, and L1 and L2 occupied different locations throughout the Sandbox container across trials. Props were used to represent the hidden object on each trial; story characters were not represented by props.

### Procedure

Each participant completed nine trials within 10 minutes. For example, in one false-belief trial, the experimenter read the first part of the story to the participant: *Sally and Ann are outside playing in the sandbox*. *Sally hides a red toy dog in the sand here* (experimenter hides dog at L1) *and then goes inside to get a drink of water*. *While Sally is inside the house*, *Ann finds the toy dog and hides it here* (experimenter moves dog to L2). Then the participant completed the visual-search filler task (*Where’s Waldo*). After 20 seconds the experimenter read the critical question: *When Sally comes back*, *where will she look for her red toy dog*? The experimenter instructed participants to respond by pointing to a particular place in the Sandbox. If participants failed to point to a particular place, the experimenter prompted them to do so.

We used the same procedures for the memory-control trials; however, when the protagonist returned, the participant was asked to report the object’s initial hiding location. For example, the corresponding memory-control question for the above story read, *Then Sally comes back*. *Where did she put her red toy dog before she went inside*? As in the false-belief trial, the correct response was L1. The false-belief and memory-control trials were methodologically identical save the critical question. Additionally, at a conceptual level, both trial types required participants to report the same information; however, the false-belief and memory-control trials required participants to access that information differently: While the false-belief questions required participants to suppress their own knowledge of L2 to respond from the naïve protagonist’s perspective, the memory-control questions only required participants to remember where the protagonist initially placed the object. Thus, *memory-control bias* arises from a failure to remember L1, whereas *false-belief bias* arises from a failure to suppress one's own knowledge and/or a failure to remember L1. Comparing the magnitude of memory-control bias to false-belief bias can reveal the extent to which these two errors account for errors in false-belief reasoning.

### Statistical analyses

We calculated bias scores as the distance in inches between L1 and the participant’s response. Bias scores approaching zero indicated that search estimates were randomly distributed around L1, where the protagonist believed the object to be (i.e., no bias on false-belief trials; accurate recall of L1 on memory-control trials). Conversely, responses in the direction of L2 indicated that the participant’s response was biased away from where the protagonist believed the object to be, toward the actual object location (i.e., bias on false-belief trials; interference from L2 on memory-control trials). Bias scores on the individual trials could fall anywhere within the Sandbox; in practice, these scores mostly occupied L1, L2, and the space between L1 and L2 (Middle responses; Table 1).

**Table 1:** Raw (percentage) response category frequencies across condition and age group.

Condition and Age Group	*L*_*1*_	Middle	*L*_*2*_
**Memory Control**			
3 yrs	17 (18.4%)	26 (28.3%)	49 (53.3%)
5 yrs	24 (30%)	12 (15%)	44 (55%)
9–12 yrs	139 (84.7%)	17 (10.4%)	8 (4.9%)
18–25 yrs	361 (80.6%)	53 (11.8%)	34 (7.6%)
65–92 yrs	197 (70.4%)	42 (15.0%)	41 (14.6%)
**False Belief**			
3 yrs	12 (13.1%)	20 (21.7%)	60 (65.2%)
5 yrs	20 (25%)	7 (8.7%)	53 (66.3%)
9–12 yrs	126 (76.8%)	11 (6.7%)	27 (16.5%)
18–25 yrs	299 (66.7%)	87 (19.4%)	62 (13.8%)
65–92 yrs	154 (55%)	51 (18.2%)	75 (26.8%)

*Note*. Response category frequencies are based on a 5% cutoff value, where 5% of the total length of the Sandbox = 3 inches. Values falling within 3 inches on either side of L1 (correct response) and L2 (incorrect response) fall into L1 and L2, respectively.

Ideally, we would have liked to maintain our original age groups (3-year olds, 5-year olds, 9- to 12-year olds, 18- to 25-year olds, and 65- to 92-year olds) to explore age variation in false-belief reasoning. However, methodological differences across samples prevented us from doing so. Specifically, because we collected our data at different times and institutions, the order in which we administered the false-belief and memory-control trials differed across samples (Table 2). Thus, comparisons of false-belief reasoning across some of the age groups were confounded by trial order (e.g., receiving the memory-control trials prior to the false-belief trials or vice versa).

**Table 2:** Trial order across age groups.

Age Group	Trial Order Number	Trial Order Condition
3- and 5-year olds	1	FB (MC), FB (MC), FB (MC), FB (MC), TB, MC (FB), MC (FB), MC (FB), MC (FB)
9- to 12-year olds, and forty-four 17- to 25-year olds	2	MC, MC, FB, FB, TB, FB, FB, MC, MC
Sixty-eight 17- to 25-year olds and the 65- to 92- year olds	3	MC, MC, FB, FB, TB, MC, MC, FB, FB

*Note*. FB = False Belief; MC = Memory Control; TB = True Belief. The parentheses in the 3- and 5-year olds trial order reflect a counterbalanced design in which the participants either received the first four trials as false-belief trials and the last four as memory-control trials or the first four trials as memory-control trials and the last four as false-belief trials.

To solve this problem, we categorized the data according to trial order rather than age. This meant that we grouped participants who received the same trial order. We analyzed age as a continuous variable within these groups. This resulted in the data being divided into the following three groups, each of which we analyzed separately: (1) 3- and 5-year olds who received Trial Order 1, (2) 9- to 12-year olds and 44 younger adults who received Trial Order 2, and (3) the remaining 68 younger adults and the older adults who received Trial Order 3. This approach allowed us to assess changes in false-belief reasoning across the preschool years, from older childhood to younger adulthood, and from younger adulthood to older adulthood, respectively. However, we were unable to assess potential changes in false-belief reasoning across groups (e.g., changes from the preschool years to older childhood).

We first completed descriptive analyses using our original categorization of age to identify the pattern of false-belief reasoning at various developmental periods. Given that prior work has demonstrated false-belief reasoning gains across the preschool years, we completed our descriptive analyses with the 3- and 5-year-old data separated. Note that trial order was counterbalanced within the preschool-aged children, thus, comparisons are not confounded by trial order. In the first set of analyses, we calculated the percentages of individual trial responses occupying L1, the Middle, and L2 in the Sandbox task (Table 1). We defined L1, Middle, and L2 responses as follows: Based on a 5% cutoff value, where 5% of the total length of the Sandbox = 3 inches, values falling within 3 inches on either side of L1 and L2 occupy L1 and L2, respectively. Middle responses were locations falling more than 3 inches (more than 5% of the Sandbox’s total length) away from L1 toward L2 and more than 3 inches away from L2 toward L1. The second analysis involved calculating the average bias scores separately on the four false-belief and four memory-control trials (Table 3). Because averaging data can obscure important details not apparent at the individual-trial level, we included the first of these analyses to show that participants of all age groups treated the Sandbox continuously rather than categorically (e.g., they chose to respond somewhere in the middle of L1 and L2 on up to 20% of 186 trials). We included the second analysis to depict the breakdown of false-belief and memory-control responses within each age group. Note that with exception to the 3- and 5-year olds, comparison of mean biases across age groups should not be made due to the previously described trial-order confound.

**Table 3:** Average false-belief bias, memory-control bias, and egocentric bias on the Sandbox task as a function of age group.

Age Group	*M* False-Belief Bias [95% CI]	*M* Memory-Control Bias [95% CI]	*M* Egocentric Bias [95% CI]
3	12.45 [11.33, 13.56]	9.98 [8.86, 11.10]	2.47 [0.57, 4.36]
5	10.69 [8.54, 12.84]	8.90 [6.74, 11.05]	1.79 [-1.22, 4.82]
9–12	1.36 [0.17, 2.55]	-0.44 [-1.00, 0.13]	1.80 [0.52, 3.07]
18–25	1.72 [1.00, 2.44]	0.32 [-0.40, 1.03]	1.40 [0.74, 2.06]
65–92	4.49 [3.50, 5.48]	1.97 [1.34, 2.60]	2.52 [1.52, 3.52]

*M* egocentric bias = false-belief bias minus memory-control bias. Higher scores indicate more bias.

Next, for each participant, we calculated a difference score (henceforth “egocentric bias,” our inverse measure of false-belief reasoning: The higher one’s egocentric bias, the lower one’s false-belief reasoning) by subtracting the average memory-control bias from the average false-belief bias (Table 3). A positive egocentric bias (false-belief bias > memory-control bias) indicated difficulty suppressing one's own knowledge to reason about another person’s false beliefs. We also considered a multiplicative measure of egocentric bias wherein we divided false-belief bias by memory-control bias; however, we believe that a difference score is a better measure of egocentric bias and by extension, false-belief reasoning, than is a ratio score. Our primary rationale is that we aimed to measure false-belief reasoning based on how much more often participants made errors in the false-belief condition relative to the memory-control condition.

To assess age variation in false-belief reasoning, we conducted a series of regression analyses assessing changes in egocentric bias across participants that received the same trial order. Thus, our three regression analyses contained age as the independent variable and egocentric bias as the dependent variable. The first regression assessed age variation in egocentric bias across the preschool years (ages 3 and 5); the second regression assessed age variation in egocentric bias from older childhood to younger adulthood (9- to 12-year olds and forty-four of the 18- to 25-year olds); the final regression assessed age variation in egocentric bias from younger to older adulthood (sixty-eight of the 18- to 25-year olds and the 65- to 92-year olds).

We conducted power analyses for each of our regression analyses with an alpha level of .05 and 1 predictor [19]). We adopted Cohen’s *f*^*2*^ as a measure of relative effect size ([20]; small effect .02 to .14; medium effect .15 to .34, and large effect ≥ .35). With *N* = 43, the regression analysis based on the preschool sample had sufficient power (.90) to detect a medium effect (*f*^*2*^ = .25). With *N* = 85, the regression analysis based on the older children and younger adult sample had sufficient power (.82) to detect a small effect (*f*^*2*^ = .10). Finally, with *N* = 138, the regression analysis based on the younger and older adult sample had sufficient power (.96) to detect a small effect (*f*^*2*^ = .10).

## Results

Table 1 reports the percentages of responses occupying L1, the Middle, and L2 in the Sandbox task. At a descriptive level, there are two notable features of these data: (1) There were fewer L1 responses (correct responses) in the false-belief condition than in the memory-control condition; (2) There were more Middle and L2 responses (incorrect responses) in the false-belief condition than in the memory-control condition.

Table 3 depicts average false-belief bias, memory-control bias, and egocentric bias as a function of age group. Importantly, and as predicted, all age groups showed more bias in false-belief than the memory-control trials, indicating that participants had more trouble suppressing their own knowledge than they did remembering the object’s initial hiding location.

Table 4 includes the results of our multiple regression analysis. Despite the small difference in mean bias scores across the preschool years (refer to Table 3), age was not a significant predictor of egocentric bias in the preschool-aged regression analysis, *F*(1, 41) = 0.13, *p* = .72, *f*^*2*^ = .003. Moreover, age was not a significant predictor of egocentric bias in the older children and younger adult regression analysis, *F*(1, 83) = 0.72, *p* = .40, *f*^*2*^ = .009. Conversely, age explained 8% of the variance in egocentric bias in the younger and older adult regression analysis, *F*(1, 136) = 11.15, *p* = .001, *f*^*2*^ = .08. Taken together, our analyses revealed similar egocentric bias across the preschool years and from older childhood to younger adulthood, followed by a small but significant difference from younger to older adulthood. The latter difference revealed that older adults showed more egocentric bias (i.e., poorer false-belief reasoning) than did younger adults.

**Table 4:** Simple regression analyses regressing egocentric bias on age.

Group/Predictor	α	β	*p*-value	*R*^*2*^
*Preschoolers (N = 43)*	3.39			.003
Age		-0.32	.72	
*Older Children and Younger Adults (N = 85)*	0.79			.009
Age		0.10	.40	
*Younger and Older Adults (N = 138)*	-0.22			.08*
Age		0.04	.001	

*Note*. Preschoolers completed Trial Order 1. Older children and 44 younger adults completed Trial Order 2. Sixty-eight younger adults and older adults completed Trial Order 3. The dependent measure was egocentric bias (false-belief bias–memory-control bias) with higher scores indicating poorer false-belief reasoning.

*p = .001.

Given that difference scores can be misleading when there are large group differences in the baseline scores that comprise the difference variable [21, 22], we completed three additional regression analyses with false-belief bias as the dependent variable and memory-control bias as an independent variable in Block 1 and age as an independent variable in Block 2. The results from our primary regression analyses held, indicating that our findings were robust to different methods of assessing false-belief reasoning.

## Discussion

We present the first study to use a single measure to assess differences in false-belief reasoning at various developmental periods across the lifespan. Using the Sandbox task, a continuous change-of-location task with multiple trials that controls for task-specific memory, we derived a measure of egocentric bias. We operationalized false-belief reasoning as the inverse of egocentric bias, where higher scores denote poorer false-belief reasoning. We observed similar false-belief reasoning across the preschool years and from older childhood to younger adulthood. The only significant age difference that we observed was from younger to older adulthood. Here, older adults exhibited more egocentric bias (i.e., poorer false-belief reasoning) than did younger adults, replicating prior work [16].

Previous studies have shown large improvements in false-belief reasoning over the preschool years, small improvements in older childhood and younger adulthood, followed by modest declines in older adulthood [6, 23, 24, 25]. Unlike the relative similarity in false-belief reasoning ability that we observed at various points across the lifespan, separate meta-analytic work in preschoolers and in adults have yielded large effects in 3- versus 5-year olds [6] and medium effects in younger versus older adults [10].

One explanation for the discrepant findings is that the field uses different tasks to study false-belief reasoning in different age groups. Most of these tasks measure what is called first-order false-belief reasoning—understanding what one person thinks (used predominantly in preschoolers) or what is called second-order false-belief reasoning—understanding what one person thinks that another person thinks (used predominantly in younger versus older adults). Moreover, many of these tasks involve dichotomous methods of assessing false-belief reasoning that fail to control for task-specific memory [26]. Conversely, our task involves a continuous method that specifically controls for task-specific memory. Unlike the single-trial classic change-of-location task, the Sandbox task can detect continuous differences in children’s and adults’ tendency to favor beliefs over reality as determinants of others’ actions [27].

The Sandbox task also can detect continuous differences in people’s ability to suppress their own knowledge when faced with conflicting perspectives [28]. For example, when analyzing performance on the classic task, researchers often omit children who cannot recall the object’s initial location (memory-control question). Performance, therefore, reflects a categorical measure of false-belief reasoning on a single trial after correcting for accurate memory (with the untested assumption that children who answer the control question correctly did not guess). In the Sandbox task, we administer multiple trials and retain all participants, but strictly control for their memory for the initial object locations. Thus, we conceptualize performance on the Sandbox task to reflect continuous false-belief reasoning after correcting for task-specific memory. Comparing these tasks, we argue that the classic task may inflate age differences: Consider a 3-year old failing the change-of-location task and a 4-year old passing the task, implying a categorical change in false-belief reasoning between the 3- and 4-year old. Conversely, our findings reveal no development in preschoolers’ false-belief reasoning, once we control for task-specific memory. Thus, the discrepancy between prior results and our results is potentially due to prior studies using tasks that fail to control for, assess (and verify) task-specific memory. Perhaps the Sandbox task’s greatest strength is that it can be used to measure variability in false-belief reasoning from preschool to old age.

The nature of classic false-belief tasks reflects the field’s original assumption that false-belief reasoning is categorical: One either understands false belief or doesn’t. Many in the field have shifted their focus to individual differences in false-belief reasoning. To study individual differences, researchers often combine performance across a battery of tasks. These combined scores transform categorical measures into a continuous composite measure. Like many developmental and cognitive psychologists, we conceive of false-belief reasoning as a continuous construct. The Sandbox task measures the continuous nature of false-belief reasoning, which is an important component of broader ToM and perspective-taking abilities. Our findings align with recent literature documenting clear age differences in various ToM skills, including false-belief reasoning, between younger and older adulthood [10, 29]. Further we provide supporting evidence for using a continuous measure of false belief to assist with delineating how and when these age differences occur and what factors may influence variability in false-belief reasoning across ages.

Aside from multiple trials and the capacity to measure a very wide age range, the Sandbox task has additional advantages over classic false-belief tasks. The Sandbox task can measure both categorical *and* continuous responses. For example, participants whose false-belief and memory-control bias scores approach the outer limits of the Sandbox task would correspond to participants who pass (zero inches) or fail (14 inches) standard categorical false-belief tasks. This is because bias scores near zero reflect L1 responses, the correct response in a two-location false-belief task. Bias scores near 14 inches reflect L2 responses, the incorrect response in a two-location false-belief task. What then do we make of bias scores that fall somewhere between zero and 14 inches, in the middle of L1 and L2? It is these middle scores that reflect the continuous nature of false-belief reasoning—the extent to which one can represent false belief. As Table 1 shows, all age groups utilized the middle of the Sandbox.

It is important to address how task demands cause continuous variation across age in the ability to demonstrate false-belief reasoning. Primary among these demands is the need to remember L1 while ignoring the highly salient and recent L2. This enables one to reason that the protagonist in the story has a false belief about where the object is located. The cognitive processes underlying these demands may be task-specific memory (for L1) and inhibitory control (ignoring L2 to reason about the protagonist’s false belief). We know that memory and inhibitory control improve from preschool to younger adulthood, followed by declines in older adulthood [30, 31, 32]. We also know that inhibitory control relates to false-belief reasoning in children and adults [33, 34]). Table 1 reveals a qualitative difference between preschoolers and older adults in our study: Preschoolers’ responses are distributed around L2 whereas older adults’ responses are distributed around L1 and the middle space between L1 and L2 in the Sandbox task. From these data, we gather that preschoolers have more trouble inhibiting their knowledge of L2 and are more prone to interference than are older adults [35].

Essentially, the Sandbox task, much like the classic change-of-location task, is a retroactive interference paradigm in which recent information (L2) interferes with one’s ability to remember older information (L1) [36]. The memory-control question allows us to control specifically for retroactive interference. The false-belief question, in addition to tapping retroactive interference, requires the extra processing step of ignoring what one knows (e.g., that Anne has moved the ball to Location 2 while Sally was away) to reason from a naïve perspective (e.g., that Sally has a false belief about where the ball is). Therefore, the false-belief question in the Sandbox task measures *whether* one can represent false belief (reflected by L1 or L2 responses), and *to what extent* one can represent false belief (reflected by middle responses).

Considering these points, we maintain that successfully reasoning about another person's false belief requires the ability to (1) represent the others' belief (which presumably children aged 4 and older, younger adults and older adults possess; younger children and infants might possess this ability too [25]), and (2) successfully represent another person's belief state while inhibiting one's own belief state in a given situation. Healthy older adults do not lose the ability to represent another person's beliefs; rather, they show poorer ability to represent another person’s beliefs *while* suppressing their own beliefs [24]. However, this process cannot simply be due to a lack of memory for basic task information, or the need to coordinate two different pieces of information (otherwise participants would have equivalent difficulty on the false-belief and memory-control questions). Rather, there seems to be something specifically difficult about the demands of simultaneously representing someone else's perspective while suppressing one's own.

### Limitations

The Sandbox task involves first-order false–belief reasoning. Thus, the results reported here may not generalize to more complex second- and higher-order tasks used to assess ToM in older children and adults, such as double bluff, faux pas, and moral judgments. Also, the current study did not assess the validity of the Sandbox task. Other work has reported significant correlations between false-belief bias in the Sandbox task and the classic change-of-location task after controlling for memory-control bias and age in preschoolers and young children [15, 37]; though inter-test correlations for ToM tasks, more generally and especially among adults, are quite low [29]. Future work might include additional age-appropriate ToM tasks of varying complexities to assess the Sandbox task’s validity as a measure of false-belief reasoning in different age groups. Other limitations to our study are that sample sizes and trial orders differed between age groups. Future work should address these limitations by obtaining large samples, assessing false-belief reasoning in different developmental periods both cross-sectionally and longitudinally, and ensuring that all participants complete the Sandbox task in the same way. This would enable more direct and continuous age comparisons across the lifespan. Future work might also explore links between false-belief reasoning and cognitive processes by including measures of language and executive function, such as inhibitory control and working memory. Finally, future work could employ formal mathematical models of the data to determine the cognitive processes associated with false-belief reasoning across the lifespan [38–40].

## Conclusions

Our results show similar false-belief reasoning abilities across the preschool years and from older childhood to younger adulthood, followed by small differences in these abilities between younger and older adults. These results highlight the relative similarity in false-belief reasoning abilities at different developmental periods across the lifespan. What our methodology and results show is that the ability to reason about someone else’s naïve mental state while ignoring one’s own knowledge (false-belief reasoning) shows only modest age-related differences across the lifespan.

## Supporting information

S1 Data Dataset used in the analysis. (XLSX)

S1 Text Description of variables in the dataset. (DOCX)
