# Supplementary material for: False-belief reasoning from 3 to 92 years of age
Source: PLoS One. 2017 Sep 28;12(9):e0185345. doi: 10.1371/journal.pone.0185345 (PMC5619768; doi:10.1371/journal.pone.0185345)
Supplement: S1 Text — (DOCX) [file pone.0185345.s002.docx]

**Description of Variables**

**Id**: Participant identification.

**Age_Group**: 1= 3-year olds, 2 = 5-year olds, 3 = 9-10 year olds, 4 = 11-12 year olds, 5 = younger adults, 6 = older adults

**Trial_Order_Reference**: Trial order received by the 3- and 5-year olds, which was as follows:
FB (MC), FB (MC), FB (MC), FB (MC), TB, MC (FB), MC (FB), MC (FB), MC (FB).
The parentheses in the 3- and 5-year olds trial order indicate that participants either received the first four trials as false-belief trials and the last four as memory-control trials or the first four trials as memory-control trials and the last four as false-belief trials. This variable is to identify the trial order received by the preschoolers (1 = first four FB trials; 0 = first four MC trials). This variable was the reference group for our trial order dummy-coded variables and thus was not entered into the regression analysis.

**Trial_Order_1:** Trial order received by school-aged children (9-10 year olds and 11-12 year olds) and 44 of the younger adults, which was as follows: MC, MC, FB, FB, TB, FB, FB, MC, MC.

**Trial_Order_2:** Trial order received by 68 younger adults and all older adults, which was as follows:
MC, MC, FB, FB, TB, MC, MC, FB, FB.

**Age_Quadratic:** Age group (using coding defined above) squared.

**FB_Avg =** Average false belief bias

**MC_Avg** = Average memory control bias

**Egocentric Bias:** Defined as Average False Belief Bias - Average Memory Control Bias

**Sandbox_Trial1:** Sandbox Story 1 raw data (L1 = 24; L2 = 10); *Note that L1 = Location 1; L2 = Location 2

**Sandbox_Trial2:** Sandbox Story 2 raw data (L1 = 18; L2 = 32)

**Sandbox_Trial3:** Sandbox Story 3 raw data (L1 = 42, 28)

**Sandbox_Trial4:** Sandbox Story 4 raw data (L1 = 36; L2 = 50)

**Sandbox_Trial5:** Sandbox Story 5 raw data (preschoolers L1 = 24; L2 = 38; all other age groups: L1 = 34; L2 = 20)

**Sandbox_Trial6:** Sandbox Story 6 raw data (L1 = 30, L2 = 44)

**Sandbox_Trial7:** Sandbox Story 7 raw data (L1 = 48, L2 = 34)

**Sandbox_Trial8:** Sandbox Story 8 raw data (L1 = 26, L2 = 40)

**Sandbox_Trial9:** Sandbox Story 9 raw data (preschoolers: L1 = 44, L2 = 30; all other age groups: L1 = 38, L2 = 24)

**Sandbox_T1_Bias:** Sandbox Story 1 raw response minus correct L1 response (24)

**Sandbox_T2_Bias:** Sandbox Story 2 raw response minus correct L1 response (18)

**Sandbox_T3_Bias:** Sandbox Story 3 raw response minus correct L1 response (42)

**Sandbox_T4_Bias:** Sandbox Story 4 raw response minus correct L1 response (36)

**Sandbox_T5_Bias:** Sandbox Story 5 (true belief) raw response minus correct L2 response (preschoolers: 38; all other age groups: 34)

**Sandbox_T6_Bias:** Sandbox Story 6 raw response minus correct L1 response (30)

**Sandbox_T7_Bias:** Sandbox Story 7 raw response minus correct L1 response (48)

**Sandbox_T8_Bias:** Sandbox Story 8 raw response minus correct L1 response (26)

**Sandbox_T9_Bias:** Sandbox Story 9 raw response minus correct L1 response (preschoolers: 44; all other age groups: 38)
